# 12. Ataxia in a child: a rare presentation of a rare entity

**DOI:** 10.1093/rap/rky033.004

**Published:** 2018-09-20

**Authors:** Swetal C Pandey, Prasan Deep Rath, Rekha Mittal, Shaloo Bhasin

**Affiliations:** 1 Rheumatology, Department of Rheumatology, Delhi, INDIA; 2 Department of Rheumatology, Max Super Speciality Hospital, New Delhi, INDIA; 3 Paediatric Neurology, Department of Paediatrics, Max Smart Hospital, Delhi, INDIA

**Introduction:** Hashimoto encephalopathy is rare type (prevalence 2.1/100000) of encephalopathy also known as steroid responsive encephalopathy. It is predominantly seen in adult female population (5: 1). In pediatric age group the mean age of presentation is 14 years with female predominance. Youngest age of onset being reported in literature is two years 10 months. It is associated with high titers of antithyroid antibodies titers. It presents with various clinical signs and symptoms. it can present as confusion, disorientation, seizure, psychosis, cerebellar ataxia to coma. In this case we are reporting a case of Hashimoto encephalopathy presenting with cerebellar ataxia and confusion in pediatric age group. with normal imaging, CSF analysis and EEG with only clue to diagnosis was raised thyroperoxidase antibody with normal thyroid function.

**Case description:** An eight year old boy presented to the emergency department in 2014 with history of slurring of speech along with difficulty in walking straight He was swaying forwards and sideways while getting up from supine position and while walking in narrow passages. It was sudden in onset. Clumsiness of hands was present in the form of illegible handwriting but not small in size. There was no history of involuntary movements nor any weakness in any form in any limb nor any sensory complaints nor any clinical history suggestive of cranial nerve involvement. He also had three to four vomiting episodes on the day of admission along with nausea, associated with headache for one week. All these complaints were never associated with fever. There was no evidence of leukocytosis in hemogram. CSF showed high number of cells with predominant lymphocytes, protein levels were slightly high with normal glucose suggesting possibility of viral meningoencephalitis. HSV PCR and varicella IGM were negative. MRI brain was normal. He was started treatment with ceftriaxone and acyclovir. Patient was discharged following which he took approximately four months, during this time his cerebellar signs gradually improved and disappeared. He visited in emergency four years later, with bilateral cerebellar signs with confusion and irritability at this time, he had fever for two to three days, two weeks back. Again hemogram, blood cultures were normal with raised inflammatory markers. This time CSF analysis was normal. MRI brain, MRI venogram both were normal. EEG was normal. Patient was started treatment with antibiotic and antiviral. Due to repeated similar episode, this time autoimmune work up was done. ANA by immunofluorescence and ANA by line immunoassay was negative along with rheumatoid factor. NMDA receptor antibody, VGKC antibody were negative along with anti NMO antibody. Thyroid function test was normal but thyroperoxidase antibodies levels were high. Diagnosis of hashimoto’s encephalopathy was made. Patient was treated with methylprednisolone pulse for three days followed by oral steroids at 1 mg/kg dose.pt showed remarkable improvement and is currently being followed up in opd regularly, steroid has been tapered slowly over five months and stopped his cerebellar signs have disappeared. His thyroperoxidase antibodies level returned to normal after four months of treatment.

**Discussion:** Hashimoto encephalopathy is rarely seen in pediatric age group, usually seen in female patients with age of onset around 14 years while our patient was male and presented at age of 8 years. Brain et al. first described case of hashimoto encephalopathy in a patient with thyroiditis and hypothyroidism, however, in many cases described patients are often euthyroid. In study by Castillo et al 65% of adults of encephalopathy, presented with cerebellar signs. In study by Enrol et al, one of four paediatric patients’ presented with gait ataxia and confusion as our patient. In children seizure was present in 66% cases. Neuropsychiatric features like confusion, restless were presenting symptoms in most of children, unlike our case it was not the presenting symptom. while there are no such reports in children. In same study we found magnetic resonance imaging was normal in all patients. Here it was found that EEG was mostly abnormal showing slowing in background rhythm, but one of four patients also showed normal EEG but this patient presented with breath holding spell and behavioural problems. Cerebrospinal analysis was done in only one patient’s of four, which was normal. Elevated anti thyroid antibodies is characteristic for Hashimoto encephalopathy. Thyroperoxidase antibody is frequently associated with hypothyroidism and hyperthyroidism, and it is present in almost all patients. Hypothyroidism was seen in 52% of the paediatric patients, and 48% were euthyroid as of our patient. All patients were treated with high dose of steroids, but in contraindication to adult patients, paediatric patients have long term sequalae. Thyroid antibodies may be elevated even with treatment although in study by enrol et al titres reduced in all patients with clinical improvement, as seen in our patient.

**Key Learning Points:** A diagnosis of Hashimoto encephalopathy is difficult to make, as it presents with varied symptoms with normal imaging, CSF analysis EEG and thyroid function test. It requires an high index of suspicion as when diagnosed patients improve almost completely with steroid. Patient presenting with neuropsychiatric manifestation in paediatric age group should also be tested for thyroid function test and anti-thyroid antibodies to rule out Hashimoto encephalopathy.

**Disclosure: S.C. Pandey:** None. **P. Rath:** None. **R. Mittal:** None. **S. Bhasin:** None.

